# Supplementary material for: Impact of Special Drying Schemes on Color Stability of Mangoes with Different Maturity Degrees
Source: Foods. 2022 Feb 23;11(5):656. doi: 10.3390/foods11050656 (PMC8909022; doi:10.3390/foods11050656)
Supplement: Supplementary file 1 [file foods-11-00656-s001.zip › foods-1567966-supplementary.pptx]

## Slide 1
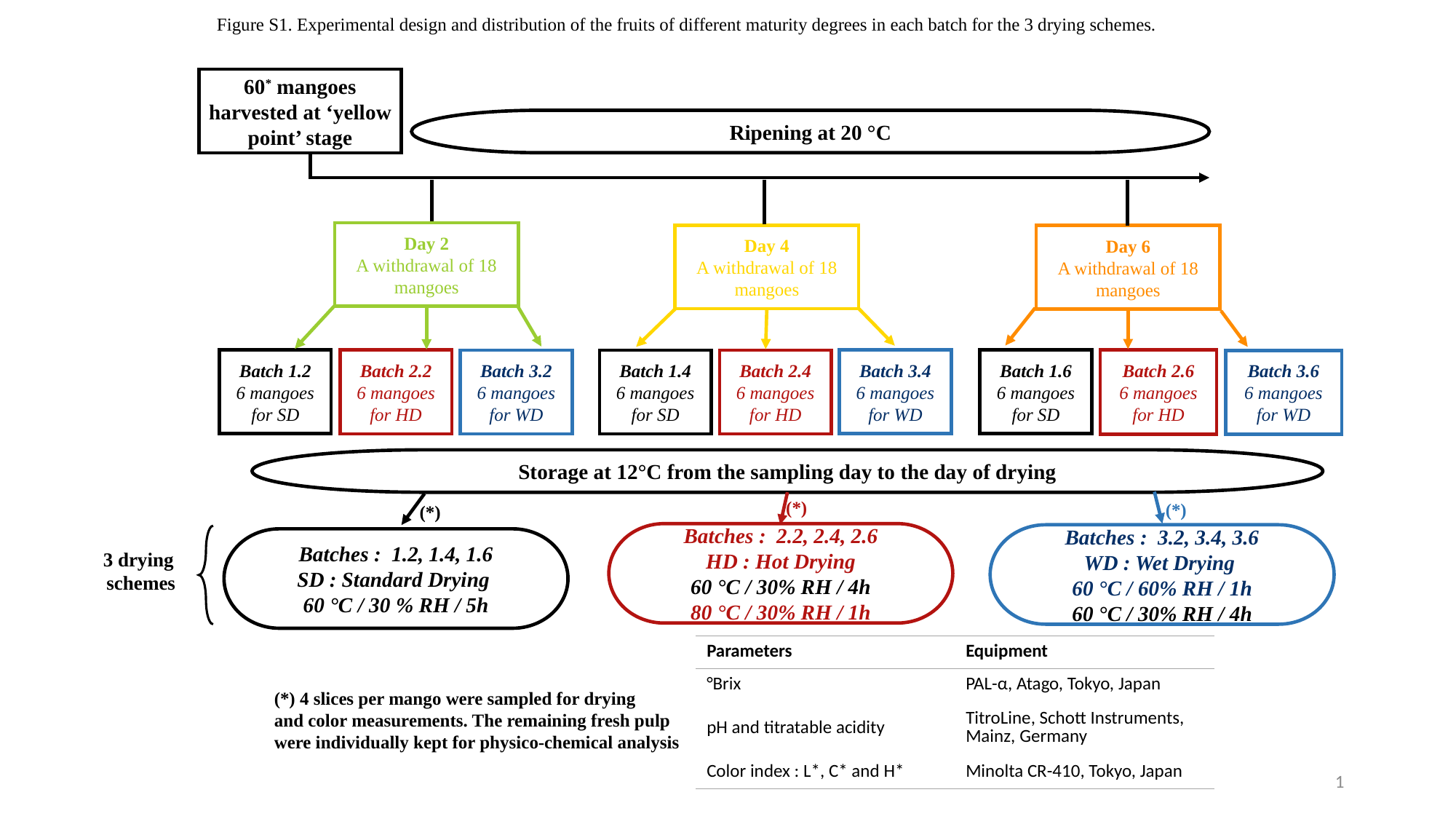

Figure S1. Experimental design and distribution of the fruits of different maturity degrees in each batch for the 3 drying schemes.
60* mangoes harvested at ‘yellow point’ stage
Ripening at 20 °C
Day 2
A withdrawal of 18 mangoes
Day 4
A withdrawal of 18 mangoes
Day 6
A withdrawal of 18 mangoes
Batch 2.6
6 mangoes for HD
Batch 1.6
6 mangoes for SD
Batch 3.4
6 mangoes for WD
Batch 1.2
6 mangoes for SD
Batch 2.2
6 mangoes for HD
Batch 3.2
6 mangoes for WD
Batch 2.4
6 mangoes for HD
Batch 1.4
6 mangoes for SD
Batch 3.6
6 mangoes for WD
Storage at 12°C from the sampling day to the day of drying
(*)
(*)
(*)
3 drying
schemes
Batches : 2.2, 2.4, 2.6
HD : Hot Drying
60 °C / 30% RH / 4h
80 °C / 30% RH / 1h
Batches : 3.2, 3.4, 3.6
WD : Wet Drying
60 °C / 60% RH / 1h
60 °C / 30% RH / 4h
Batches : 1.2, 1.4, 1.6
SD : Standard Drying
60 °C / 30 % RH / 5h
| Parameters | Equipment |
| --- | --- |
| °Brix | PAL-α, Atago, Tokyo, Japan |
| pH and titratable acidity | TitroLine, Schott Instruments, Mainz, Germany |
| Color index : L\*, C\* and H\* | Minolta CR-410, Tokyo, Japan |
(*) 4 slices per mango were sampled for drying
and color measurements. The remaining fresh pulp were individually kept for physico-chemical analysis
1

## Slide 2
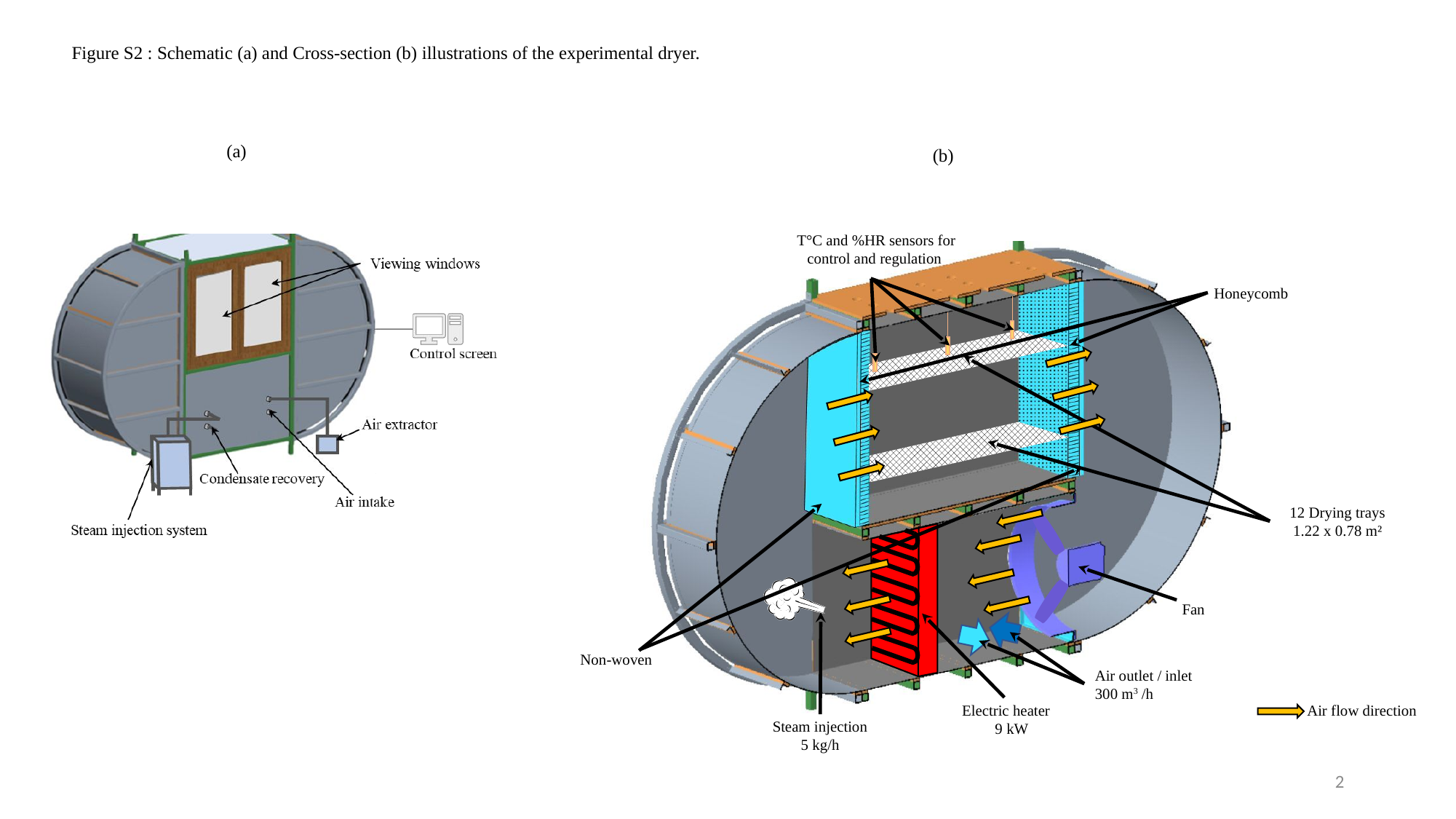

Figure S2 : Schematic (a) and Cross-section (b) illustrations of the experimental dryer.
(a)
(b)
T°C and %HR sensors for
control and regulation
Honeycomb
12 Drying trays
1.22 x 0.78 m²
Fan
Non-woven
Air outlet / inlet
300 m3 /h
Electric heater
9 kW
Steam injection
5 kg/h
Air flow direction
2
